# Molecular Basis of Filamin A-FilGAP Interaction and Its Impairment in Congenital Disorders Associated with Filamin A Mutations

**DOI:** 10.1371/journal.pone.0004928

**Published:** 2009-03-18

**Authors:** Fumihiko Nakamura, Outi Heikkinen, Olli T. Pentikäinen, Teresia M. Osborn, Karen E. Kasza, David A. Weitz, Olga Kupiainen, Perttu Permi, Ilkka Kilpeläinen, Jari Ylänne, John H. Hartwig, Thomas P. Stossel

**Affiliations:** 1 Translational Medicine Division, Department of Medicine, Brigham and Women's Hospital, Harvard Medical School, Boston, Massachusetts, United States of America; 2 Laboratory of Organic Chemistry, Department of Chemistry, University of Helsinki, Helsinki, Finland; 3 Department of Biological and Environmental Science, University of Jyväskylä, Jyväskylä, Finland; 4 Department of Physics & SEAS, Harvard University, Cambridge, Massachusetts, United States of America; 5 Program in Structural Biology and Biophysics, Institute of Biotechnology, University of Helsinki, Helsinki, Finland; University of Birmingham, United Kingdom

## Abstract

**Background:**

Mutations in filamin A (FLNa), an essential cytoskeletal protein with multiple binding partners, cause developmental anomalies in humans.

**Methodology/Principal Findings:**

We determined the structure of the 23^rd^ Ig repeat of FLNa (IgFLNa23) that interacts with FilGAP, a Rac-specific GTPase-activating protein and regulator of cell polarity and movement, and the effect of the three disease-related mutations on this interaction. A combination of NMR structural analysis and *in silico* modeling revealed the structural interface details between the C and D β-strands of the IgFLNa23 and the C-terminal 32 residues of FilGAP. Mutagenesis of the predicted key interface residues confirmed the binding constraints between the two proteins. Specific loss-of-function FLNa constructs were generated and used to analyze the importance of the FLNa-FilGAP interaction *in vivo*. Point mutagenesis revealed that disruption of the FLNa-FilGAP interface perturbs cell spreading. FilGAP does not bind FLNa homologs FLNb or FLNc establishing the importance of this interaction to the human *FLNa* mutations. Tight complex formation requires dimerization of both partners and the correct alignment of the binding surfaces, which is promoted by a flexible hinge domain between repeats 23 and 24 of FLNa. FLNa mutations associated with human developmental anomalies disrupt the binding interaction and weaken the elasticity of FLNa/F-actin network under high mechanical stress.

**Conclusions/Significance:**

Mutational analysis informed by structure can generate reagents for probing specific cellular interactions of FLNa. Disease-related FLNa mutations have demonstrable effects on FLNa function.

## Introduction

Filamin A (FLNa), encoded in humans and mice by a gene on the X chromosome, is an abundant and ubiquitously expressed non-muscle isoform of a family of actin cross-linking proteins [1]. Human melanoma cells lacking FLNa protein have unstable plasma membranes, do not polarize or undergo locomotion, and lack functional readouts for many of the identified FLNa-binding partners, but restoring normal levels of FLNa in these deficient cells rescues these functions [2]–[4]. Mutations of the *FLNa* gene were first identified in human periventricular nodular heterotopia (PVNH), an X-linked neuronal migration disorder that predominantly affects females and results in embryonic lethality in hemizygous males [5]. *FLNa* mutations are also associated with a group of X-linked skeletal anomalies including frontometaphyseal dysplasia (FMD) and cardiovascular defects such as familial cardiac valvular dystrophy, the most common indication for valvular surgery [6]–[8]. Complete loss of Flna in mice results in embryonic lethality with bleeding and cardiovascular malformations [9], [10]. This wide range of phenotypes is presumably attributed to alterations of FLNa association with F-actin and its binding partners, obstructing analysis of mechanisms underlying FLNa pathogenesis.

FLNa is a dominant isoform of FLN family proteins (a, b and c) and all isoforms are dimers of 270∼280 kDa subunits that have N-terminal spectrin-related actin-binding domains (srABD) separated from C-terminal dimerization domain by 23 Ig repeats organized as linear rod like strands. Two flexible hinges separate Ig repeats 15 and 16 and 23 and 24 [1], [11]. FLNa cross-links F-actin to form orthogonal networks that are responsible for cellular integrity and mechanics and attaches to membrane receptors including adhesion molecules and ion channels. FLNa is also a scaffold for numerous intracellular signaling intermediates. One of these, FilGAP, has a pleckstrin homology domain for membrane lipid binding, a GTPase-activating protein (GAP) domain, and a coiled-coil domain responsible for FLNa binding [12]. FilGAP specifically inactivates Rac *in vivo*, and this activity requires its FLNa association. FilGAP controls cell polarity and movement downstream of ROCK (Rho-kinase) [12].

Sorting out the contributions of these numerous interactions to *in vivo* function requires structural information to enable use of point mutant FLNa or partners lacking specific activities that are otherwise fully functional. Here we describe the structure of the FLNa/FilGAP complex and use the information to engineer mutant protein incapable of expressing FilGAP function *in vivo*. We also show that FilGAP interacts exclusively with FLNa among its homologs and specific disruption of the FLNa-FilGAP interaction perturbs cell spreading. Furthermore, we show that two FLNa mutations found in PVNH and FMD patients disrupt the folding of FLNa Ig repeat (IgFLNa) 23 and abolish FilGAP binding, establishing a link between downstream signaling of Rac and the patients' pathogenesis.

## Results

### Identification of the FLNa-binding interface with FilGAP

**Figure 1:**
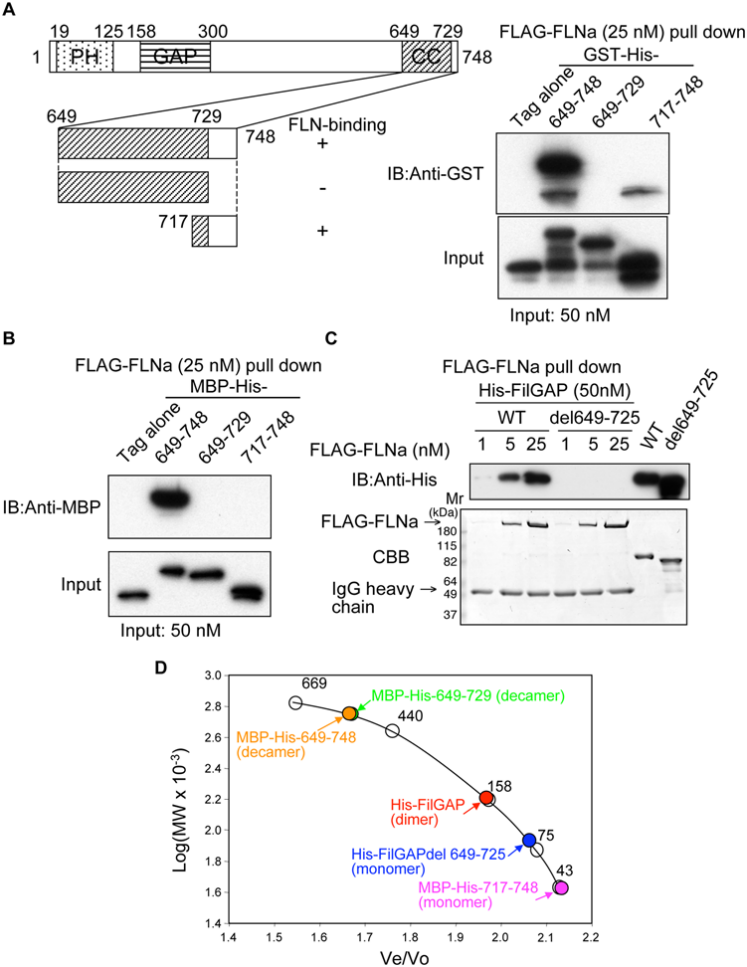
Localization of FLNa-FilGAP binding site. (A) Schematic representation of FilGAP and its truncation series. The pleckstrin-homology (PH), GTPase-activating protein (GAP), and coiled-coil (CC) domains predicted by EMBnet COILS are shown. Right panel shows binding of FLAG-FLNa to GST-FilGAP fragments illustrated in the left panel. Their interactions were analyzed by pull-down using FLAG-specific mAb immobilized on beads. Bound protein was detected by immunoblotting using rabbit pAb to GST. (B) FilGAP fragments were fused to MBP-His-tag and their binding to FLAG-FLNa were analyzed by pull-down using FLAG-specific mAb immobilized on beads. Bound protein was detected by immunoblotting using rabbit pAb to MBP (C) His-tag FilGAP, or FilGAP lacking residues 649–725 (50 nM), were mixed with increasing amounts of FLAG-FLNa and immunoprecipitated with FLAG-specific mAb immobilized on agarose. Bound FilGAP was detected by immunoblotting using anti-His-tag mouse mAb conjugated with horse radish peroxidase (upper panel). The lower panel shows proteins visualized by CBB staining. (D) Molecular weight calibration curve obtained with a Superose 6 10/300 gel filtration column. Molecular size standards (open circle) used were thyroglobulin (669 kDa), ferritin (440 kDa), aldolase (158 kDa), conalbumin (75 kDa), and ovalbumin (43 kDa). Colored circles indicate the sizes of His-FilGAP, His-FilGAP lacking residues 649–725 or FilGAP truncates fused to MBP-His-tag.

Most of the protein constructs used for *in vitro* binding assay were obtained in good yield and purity (Figure S1A). Figure 1A shows a schematic diagram of FilGAP structure and demonstrates that the C-terminal 100 residues (649–748 amino acid, aa) of FilGAP tagged to a glutathione S-transferase-hexahistidine (GST-His) interact with purified full-length FLNa *in vitro*, consistent with previous results [12]. Further deletion constructs identified that the last 32 residues (717–748aa, FilGAPC32), but not residues 649–729 of the predicted coiled-coil domain by EMBnet COILS, are the FLNa-binding site (Figure 1A). FilGAPC32 tagged to a maltose binding protein followed by hexahistidine (MBP-His), however, did not pull down with FLAG-FLNa (Figure 1B), and a synthetic peptide of FilGAPC32 immobilized on Sepharose also did not precipitate FLNa (data not shown). Analytical gel filtration demonstrated that full-length FilGAP is a dimer and FilGAP lacking residues 649–725, which contains the minimum FLNa-binding site (residues 726–734, see below), is a monomer (Figure 1D and Figure S1B) and does not form a tight complex with FLNa (Figure 1C). MBP-His-FilGAP constructs containing residues 649–729 eluted as a decamer from gel filtration columns, whereas MBP-His-FilGAPC32 was monomeric (Figure 1D and Figure S1B).

**Figure 2:**
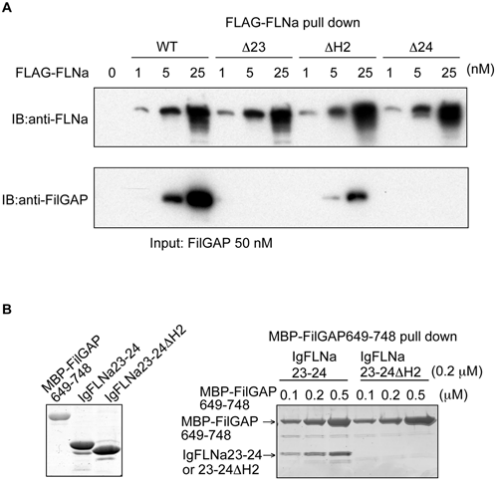
FLNa dimerization and hinge-2 are essential for high avidity binding to FilGAP. (A) Full-length FilGAP was pulled down with increasing amounts of wild-type and deletion mutants (Ä23; deletion of IgFLNa23, ÄH2; deletion of FLNa hinge-2, Ä24; deletion of IgFLNa24) of FLNa tagged to FLAG immunoprecipitated with FLAG-specific mAb immobilized on agarose. Bound FilGAP was detected by immunoblotting using rabbit pAbs to FilGAP. (B) Left panel shows purified MBP-FilGAP649-748, IgFLNa23-24, and IgFLNa23-24 ÄH2 separated on SDS-PAGE and stained with CBB. Right panel; IgFLNa23-24 or IgFLNa23-24 ÄH2 were pulled down with amylose beads coated with increasing amounts of the MBP-FilGAP649-748. Proteins were visualized by CBB staining.

FLAG-tagged full-length FLNa pulled down full-length FilGAP *in vitro*, and deletion of FLNa Ig repeat 23 abolished this interaction (Figure 2A), consistent with previous results [12]. The interaction was diminished or abolished respectively by deletion of hinge 2 and FLNa Ig repeat 24 (Figure 2A). Deletion of the hinge 2 from the C-terminal of IgFLNa23-24 disrupted FilGAP binding (Figure 2B). A hinge 2 plus IgFLNa24 construct does not bind FilGAP (data not shown), indicating that hinge 2 is not the FilGAP-binding site. Electron micrographs of mutant FLNa constructs demonstrate that their shapes are indistinguishable from wild-type FLNa (Figure S2A), and these mutations do not affect FLNa's actin gelation activity (data not shown).

### Solution structure of IgFLNa23 and mapping of the FilGAP binding site

**Figure 3:**
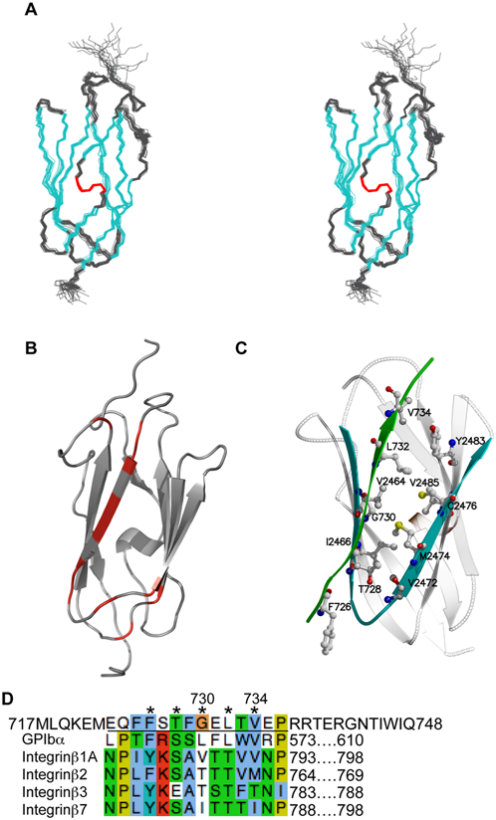
The structure of IgFLNa23 and location of the FilGAP binding interface. (A) A stereo image of superimposed backbone traces of the 20 structures in NMR structure ensemble of IgFLNa23. Cyan and red colors indicate β-strand and α-helical elements, respectively. (B) FilGAP binding interface revealed by the chemical shift changes in the ^15^N-HSQC NMR spectrum of IgFLNa23. The signals that disappeared or divided upon peptide addition are indicated in red on IgFLNa23. (C) The model of FilGAP peptide (green strand) interaction based on the IgFLNa17-GPIbα complex structure. Residues mutated in this study are indicated. (D) The sequence alignment of the last 32 residues of FilGAP and FLNa-binding sites of GPIbá and â-integrins. The alignment was generated using ClustalW program. The underline indicates the peptide used for NMR titration. Amino acids indicated with asterisks face a glove generated between the C and D strands of the IgFLNa domain which are mainly involved in binding interaction.

To map the binding site between IgFLNa23 and the C-terminal peptide of FilGAP more closely, we first utilized solution state NMR spectroscopy. All backbone amide signals were visible in the ^15^N-HSQC spectrum and nearly complete ^1^H-, ^13^C- and ^15^N-resonance assignment of IgFLNa23 was achieved. Structure determination yielded a structure of excellent quality. Almost all residues reside on the favored areas of the Ramachandran plot and the root mean square deviation (RMSD) values of the structure family are very low (Table S1). The structure of IgFLNa23 (Figure 3A) has a traditional Ig-like fold containing two β-sheets with four β-strands in each. The structure is very similar to the crystal and solution structures of IgFLNc23 (PDB accession codes 2nqc and 2d7q) [13] and the solution structure of IgFLNb23 (PDB accession code 2eec), and the RMSD of the best possible alignment to these structures ranged from 0.7 to 1.2 Å.

The binding site of FilGAP was mapped by following chemical shift changes in the ^15^N-HSQC NMR spectrum of IgFLNa23 upon addition of a synthetic FilGAP14 peptide, ^723^EQFFSTFGELTVEP^736^. This shorter peptide was used instead of FilGAPC32 because it was more soluble and thus better suited for NMR studies. The interaction between IgFLNa23 and FilGAP14 was in the fast-to-intermediate exchange region. Large excess of peptide was needed to obtain clear changes in the ^15^N-HSQC spectrum, suggesting that the affinity of IgFLNa23-FilGAP14 interaction is weak. Many signals of IgFLNa23 disappeared or divided into multiple peaks in the course of FilGAP14 titration, which made tracking of the chemical shift changes difficult (Figure S3). The disappearance and division of the signals was considered to be an indication of close proximity to the binding site. All affected peaks are located close to the CD-interface of IgFLNa23 (Figure 3B). Due to weakness of the IgFLNa23-FilGAP14 interaction NMR spectroscopic structure determination of the complex turned out to be unfeasible.

### *In silico* model of the IgFLNa23-FilGAP complex

The CD faces of IgFLNa domains are common binding sites in other known filamin interactions, including platelet glycoprotein (GP) Ibα binding to IgFLNa17 [14] and integrin β subunit cytoplasmic tail binding to IgFLNa21 [15], [16]. As FilGAP also interacted with the CD face of IgFLNa23, and the amino acid sequence of FilGAPC32 could be aligned to the β-strand forming filamin-interacting peptides of GPIbα and integrins (Figure 3D), we modeled the FilGAPC32-IgFLNa23 interaction based on the complex between IgFLNa17 and a GPIbα peptide (Figures 3C and S4).

**Figure 4:**
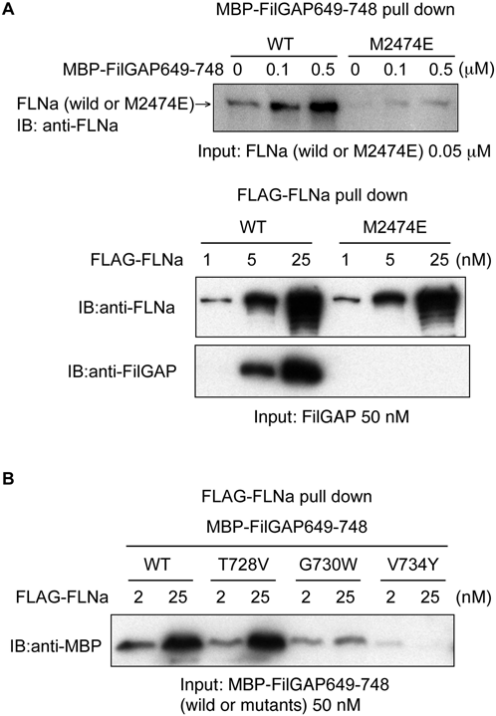
Point mutations of FLNa and FilGAP confirms the in silico model of their binding interaction. (A) A point mutation in FLNa (M2474E) abolishes the complexing of FLNa and FilGAP. The upper panel shows amylose beads coated with MBP-FilGAP649-748 pulls down wild-type FLNa but not FLNaM2474E. The lower panel shows wild-type (WT) FLAG-FLNa immobilized on FLAG-specific mAb immobilized on agarose beads, but not FLAG-FLNaM2474E, pull down full-length FilGAP. (B) FLAG-FLNa does not pull point mutants of FilGAP at G730W and V734Y. T728V mutation has no effect on the interaction.

To verify that the interaction site with FilGAP is on the CD face of IgFLNa23 we next mutated the hydrophopic M2474 to negatively charged glutamate. Indeed, the point mutation M2474E in IgFLNa23 abolished the interaction of full length FLNa with recombinant FilGAP C-terminal fragment and with full length FilGAP *in vitro* as predicted (Figure 4A). However, the mutant filamin fully retained F-actin gelation activity, and its morphology is indistinguishable from wild-type FLNa in electron micrographs (Figure S2). NMR spectra also showed that M2474E IgFLNa23 was fully folded (Figure S5). Thus, this mutation further confirms that also in the context of full length FilGAP and FLNa, the major FilGAP binding site is at the CD face of IgFLNa23.

To confirm the position of the β-strand forming segment of FilGAP we mutated G730 and V734 to bulky aromatic residues. G730W and V734Y substitutions perturbed the interaction of FilGAP C-terminal fragments with the full-length FLAG-tagged FLNa, conforming that these residues are indeed oriented towards the interface (Figure 4B). The effect of V734Y substitution also suggests that we modeled the β-strand interface in correct position of FilGAP, since in our model this residue at the end of the β-strand. The point mutation T728V had no effect in the interaction (Figure 4B).

### Effect of FLNa-FilGAP interaction on cell spreading

**Figure 5:**
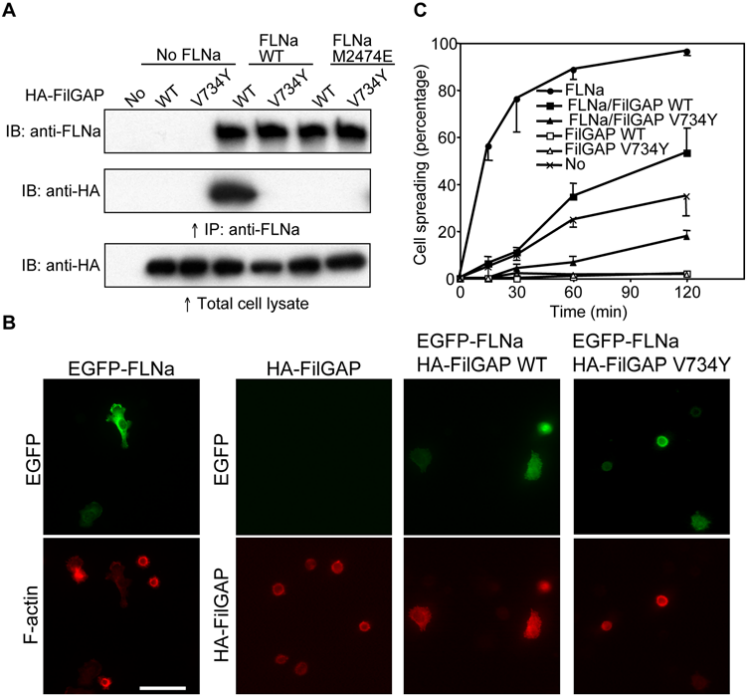
In vivo interaction of FLNa and FilGAP. (A) Immunoprecipitation of the FLNa/FilGAP complex expressed in FLNa-deficient M2 cells. Wild-type or mutant (M2474E) FLNa were co-expressed with wild-type or mutant (V734Y) HA-tagged FilGAP in M2 cells. FLNa was immunoprecipitated with FLNa-specific mAb and bound HA-FilGAP was detected by immunoblotting using HA-specific mAb. (B and C) Effects of wild-type and mutant FilGAP on cell spreading. M2 cells were transfected with EGFP-FLNa, wild-type or V734Y mutant HA-FilGAP. After 48 h, cells were trypsinized, plated on coverslips coated with fibronectin, and fixed at 15, 30, 60, and 120 min after plating. Cells were stained with mouse mAbs to HA for FilGAP (red, three lower right panels) or Texas-red phalloidin for F-actin (red, lower left panel). FLNa was detected with EGFP (green, upper panels). Stained cells at 120 min after plating are shown in (B). Scale bar, 50 ìm. The percentages of spread cells were plotted as the mean±SEM (n = 3) in (C).

To study the effects of FLNa and FilGAP mutations *in vivo*, full length proteins were expressed in filamin-deficient M2 cells. Point mutations of FLNa(M2474E) and FilGAP(V734Y) confirm that these residues are critical for the binding partnering interaction *in vivo* (Figure 5A). Transfection of either wild-type or V734Y mutant hemaglutin (HA)-FilGAP in FLNa-null M2 cells suppressed spreading on fibronectin-coated coverslips (Figure 5, B and C). Co-expression of EGFP-FLNa with wild-type FilGAP diminished the effect of FilGAP, but expression of the mutant FilGAP did not have this effect (Figure 5, B and C).

### FilGAP specifically interacts with FLNa

**Figure 6:**
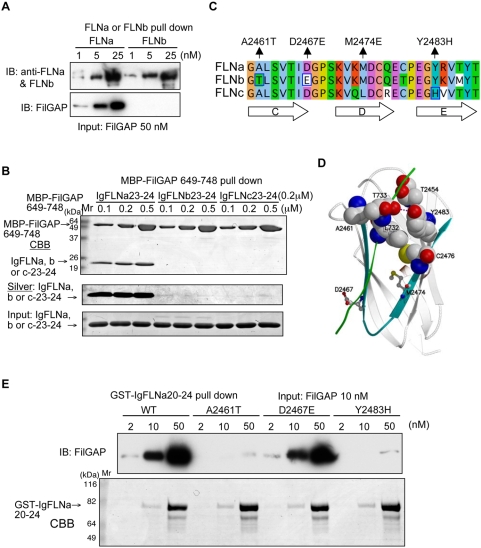
FilGAP specifically interacts with FLNa isoform. (A) Full-length FLNa, but not FLNb, pulls down FilGAP. Increasing amounts of either FLNa or FLNb were incubated with FilGAP and immunoprecipitated with mAbs to FLNa or FLNb. Bound FilGAP was detected by immunoblotting using rabbit pAbs to FilGAP. (B) MBP-FilGAP649-748 specifically binds the C-terminal of FLNa, but not FLNb or FLNc. Equal amounts of repeats 23–24 of FLNa, b, or c (0.2 ìM) were pulled down with increasing amounts of MBP-FilGAP659-748. Proteins were visualized by CBB staining (top and bottom). The top CBB-stained gel was destained and restained with silver (middle). (C) Sequence alignment of the C–E strands of the IgFLN23 isoforms. FLNa A2461T, M2474E and Y2483H point mutants do not interact with FilGAP as shown in Figures 4A, 5A and 6E. (D) Model of the IgFLNa23-FilGAP complex. Residues mutated in this study and some critical residues for their interaction are indicated. The purple dotted line shows the possible stabilizing hydrogen-bond between Tyr2483 and Thr733. (E) A point mutation of FLNa at Ala2461 to Thr or Tyr2483 to His are sufficient to abolish the complexing of FLNa and FilGAP. Full-length FilGAP (input: 10 nM constant) was pulled down with increasing amount of GST-IgFLNa20-24 immobilized on glutathione beads in a dose-dependent fashion. Mutations corresponding to A2461T or Y2483H in FLNa, but not D2467E, disrupt FilGAP binding. Bound FilGAP was detected by immunoblotting using rabbit pAbs to FilGAP. GST-FLNa constructs were detected by CBB staining.

Although the overall structure of IgFLNa23 is similar to those of IgFLNb23 and IgFLNc23, immunoprecipitation of full-length FLNb did not pull down full-length FilGAP *in vitro* (Figure 6A) and the C-terminal of FilGAP interacted only with the C-terminal of FLNa, not FLNb and c (Figure 6B). There are two amino acid changes in the CD strands of FLNa versus FLNb: Ala2461 in FLNa is substituted to Thr in FLNb and Asp2467 is Glu in FLNb. (Figure 6C and D). A mutagenetic analysis demonstrated that the A2461T substitution explains the specificity between FLNa and FLNb (Figure 6E). It is likely that the principal interactions between FLNb (and FLNa∶A2461T) and FilGAP are disturbed by the close proximity of two threonine side chains, since threonine is a bulky residue adjacent to the main chain (in Figure 6D wild-type FLNa is shown). *In silico* modeling suggests that amino acid residues comprising the CD strands of IgFLNc23 do not define the different specificity of FLNa and FLNc. Instead, substitution of Y2483, which is involved in hydrophobic contacts with FilGAP (Leu732 ) and surrounding amino acids in FLNa (Thr2454, Cys2476) to histidine alters the interaction (Figure 6D). It is also possible that Y2483 could donate a hydrogen-bond to the main-chain oxygen of T733 in FilGAP, thus, stabilizing FilGAP binding further. Such an interaction is not possible with a histidine in position 2483. *In vitro* pull down experiments confirmed this prediction (Figure 6E).

### Effects of disease-related mutations of FLNa on its cross-linking activity and FilGAP binding

**Figure 7:**
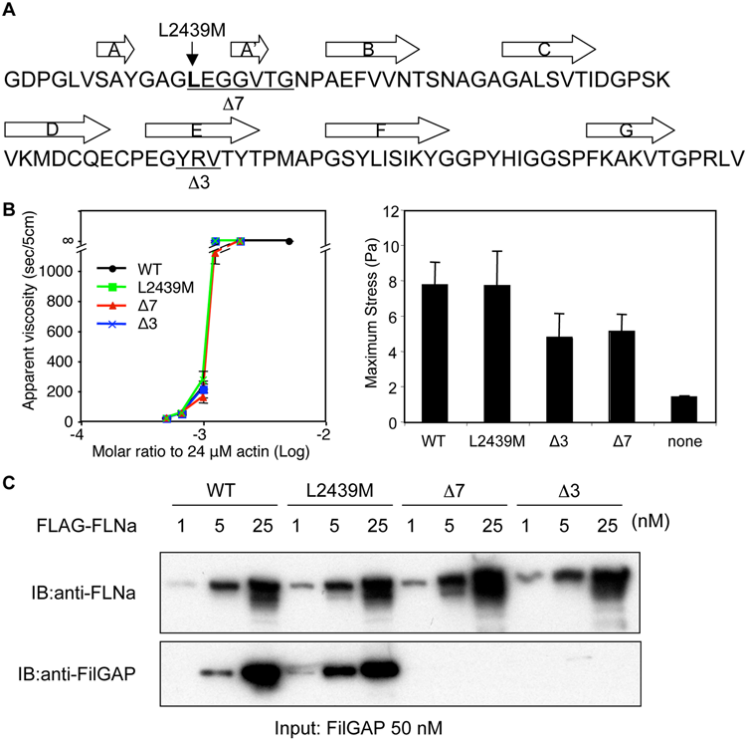
Effect of disease-related mutations in repeat 23 of FLNa on FilGAP binding. (A) Amino acid sequence of human IgFLNa23. Point (L2439M) and deletion (Ä7 and Ä3) mutants of FLNa are linked to human diseases and shown in underline. Box arrows indicate â-sheet strands. (B) Rheological properties of the FLNa mutants. Left panel, Apparent viscosity of 24 ìM actin polymerized with the indicated molar ratios of wild-type and mutant FLNa. Error bars indicate SEM from triplicates. Right panel, Maximum shear stress supported by 12 ìM F-actin polymerized with or without 0.06 ìM wild-type and mutant FLNa. Error bars indicate s.d. from triplicates. The linear and nonlinear rheological properties are provided in (Figure S6). (C) *In vitro* binding of recombinant human FLAG-FLNa and FilGAP purified from Sf9 insect cells. FilGAP was incubated with increasing amounts of wild-type or mutant FLNa and the complex was co-immunoprecipitated with FLAG-specific mAb immobilized on agarose beads. Bound proteins were detected by immunoblotting.

A single *de novo* mutation, 7315C>A of the FLNA gene, which results in PVNH and FMD in females, leads to two transcripts, an L2439M point mutant and a deletion mutant lacking 7 amino acids (Δ7: L2439-G2445) within repeat 23 (Figure 7A) [6], [7]. Another *de novo* mutation in repeat 23 is 7447del9 (or Δ3: Y2483-V2485, Figure 7A) and is associated with FMD in heterozygous females [6]. We tagged these transcripts with EGFP at their N-termini and expressed them in M2 cells with the same transfection efficiency as wild-type FLNa, but observed no obvious differences in subcellular distribution. The expressed proteins were stable *in vivo* at least up to 72 h (data not shown). FLAG-tagged-FLNa mutants were expressed in sf-9 cells and purified in good yield (Figure S1A). These mutations do not affect dimerization and gelation activity of FLNa, although the Δ7 mutation slightly diminished gelation activity (Figure 7B and Figure S2). Quantitative rheological measurements of F-actin/FLNa networks demonstrated that the L2439M point mutation does not affect linear elasticity, maximum stress, or maximum nonlinear stiffness, whereas Δ7 and Δ3 mutations diminished both the maximum stress and maximum nonlinear stiffness, leading to networks that rupture more easily under external mechanical stress (Figure 7B and Figure S6). Figure 7C demonstrates that the Δ7 and Δ3 mutations completely eliminated FLNa interaction with FilGAP, while no effect on FilGAP binding was detected with a point mutation at L2439M. In line with these results L2439M IgFLNa23 was fully folded when expressed alone in *E. coli* and the NMR spectrum showed changes only near the mutated residue (Figure S7). No NMR spectra of Δ7 and Δ3 IgFLNa23 could be obtained due to sample aggregation, suggesting that these deletions disrupt folding of the domain.

### Structure of the FLNa-FilGAP complex

**Figure 8:**
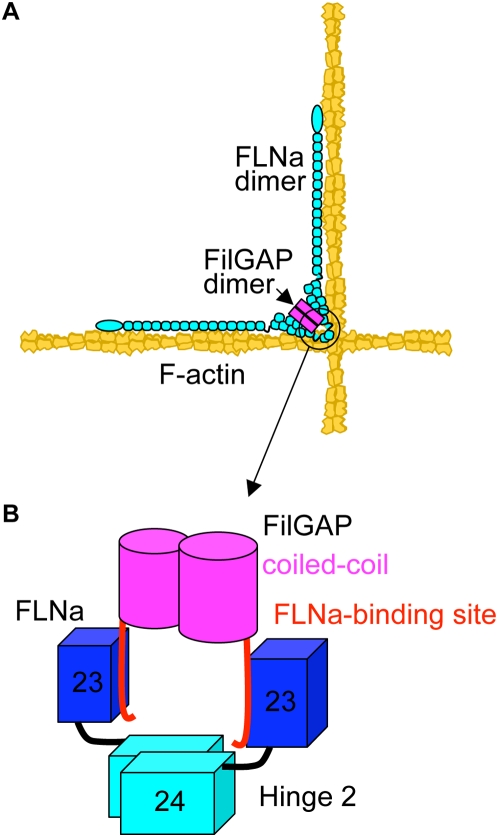
Model of the FLNa-FilGAP complex. (A) Schematic diagram of geometry of the FilGAP/FLNa/F-actin complex, suggesting mechanical regulation of FilGAP binding to FLNa by shear stress to FLNa/F-actin networks. (B) Schematic diagram of the proposed structure of the C-terminal domains of the FLNa-FilGAP complex. Atomic structure of the IgFLNa23 (blue) complexed with FilGAP peptide (red) is shown in Figure 3C. FLNa and FilGAP are dimerized through repeat 24 (cyan) and coiled-coil domain (magenta), respectively, contributing high avidity binding. The hinge-2 (black) accommodates proper geometry of their binding interface.

Since both the full-length FLNa and FilGAP are prone to aggregate at high concentrations requisite for crystallization (>2 mg/ml, data not shown), electron microscopy was used to study their structure at low concentrations (Figure S8). Purified FilGAP molecules appeared to be flexible dumbbells with two globules (∼14.2+/−2.05 nm diameters; n = 50) orienting at various angles. The mixture of FLNa and FilGAP demonstrated that the globules attached to the C-termini of FLNa molecules, consistent with the biochemical findings, whereas FLNaM2474E did not complex with FilGAP. Since gel-filtration revealed that FilGAP molecules are dimers, each globule corresponds to one FilGAP molecule (Figure 8 and Figure S8).

## Discussion

We have mapped the binding site between the Rac-specific GTPase-activating protein FilGAP and FLNa. We showed that the residues 723–736 interact with the CD face of IgFLNa23. Biochemical analysis indicated that dimerization of both FLNa and FilGAP and FLNa hinge 2 are required for effective interaction. Furthermore we showed that FilGAP is the first interaction partner that is specific to FLNa, as no binding to FLNb or FLNc was observed. Site-directed mutagenesis revealed that abolishing the FLNa-FilGAP interaction perturbs cell spreading. Disease-causing mutations that disrupt the folding of IgFLNa23 also obliterated FLNa-FilGAP interaction.

### The FLNa-binding site of FilGAP is followed by the coiled-coil domain

We previously mapped the FLNa-binding domain of FilGAP to its C-terminal 100 residues (649–748aa) which were predicted to form a coiled-coil structure [12]. Although EMBnet COILS program predicts three alternative coiled-coil segments (649–725, 648–729, 645–735), the biochemical data suggests that the minimum FLNa-binding site is not a coiled-coil because the last 32 residues (717–748) do not dimerize, and FilGAP lacking residues 648–725 is monomeric. Hence, these data indicate that the FLNa interaction site on FilGAP is outside of the coiled coil region and is available for the FLNa binding.

### FilGAP interacts with the CD face of IgFLNa23

NMR titration experiments showed that FilGAP14 peptide (residues 723–736) interact with the CD-face of IgFLNa23. This interaction sequence is similar to the β-strand forming peptides of platelet GPIbα and integrins β1, β2, β3 and β7 that interact with the corresponding faces of IgFLNa17 and IgFLNa21 [14]–[16]. In the binding interface between FLNa and FilGAP, the amino acids indicated with asterisks in Figure 3D (Phe726, Thr728, Gly730, Leu732, and Val734) face the groove formed between the C and D strands of the IgFLNa repeat, similar to the binding interaction of GPIbα and the β-integrins with FLNa. Further evidence for FilGAP's use of a similar binding motifs are: 1) Phe726, Leu732, and Val734 in the FilGAP binding site are identical to the corresponding amino acids of GPIbα, which make strong hydrophobic contacts with FLNa [14]; and 2) the amino acid positions corresponding to Thr728 and Gly730 in FilGAP vary between GPIbα and integrin β. Serine occupies this position in GPIbα, and in most of the integrin β chains, and fits within the FilGAP-FLNa interface. The small size of Gly730 would not be expected to disrupt binding.

Based on the NMR analysis and our previous structures of IgFLNa17-GPIbα and IgFLNa21-integrin β7 complexes, we proposed a model for the interaction, and we verified it by introducing point mutations in both repeat 23 of full length FLNa and in FilGAP. The effects of G730W and V734Y mutations on the interaction suggest that these residues point towards the interface, as predicted in our model (Figure 3C). Because the P736 residue is at the end of modeled β strand, alignments where the peptide would be slipped backwards are not possible (Figure 3D). Alternatively, slipping the peptide forward would bring V734 to a position where mutation would not affect the interaction.

### Higher order structure of the FLNa-FilGAP complex

Complexing of FilGAP with FLNa is not simply defined by the binding interface, because the interaction is diminished by deletions of 1) dimerization domain IgFLNa24, 2) FLNa hinge 2, and 3) FilGAP coiled-coil domain. The coiled-coil domain of FilGAP is a decamer when fused to an MBP-His tag, presumably because coiled-coil domains aggregate in a variety of conformations [17]. However, full-length FilGAP and FilGAP lacking its coiled-coil domain (residues 649–725) elute as a dimer and a monomer, respectively, on a gel filtration chromatography. Residues 649–729 do not bind FLNa, indicating that the coiled-coil interaction functions only to dimerize FilGAP. These data also suggest that FilGAP domains other than the coiled-coil domain allow dimerization while preventing oligomerization. Although NMR detects interaction of IgFLNa23 with FilGAP peptide, neither monomeric IgFLNa23 nor FilGAP biochemically pulled each other down, indicating that their affinities for each other are very low. Binding strength or avidity of macromolecules is governed by three major factors: the intrinsic affinity of the individual binding interface, the valency of the binding site(s), and geometric arrangement of the interacting components [11], [18]. We have previously shown that dimerization of FLNa increases its affinity for F-actin by at least one order of magnitude. Considering that the apparent dissociation constant of FLNa-FilGAP of ∼0.2 µM requires (assessed from ref. [11]), FLNa and FilGAP each to be dimers, and IgG antibody molecules bind ∼1000 times more strongly than monovalent Fab subfragments to a antigen [18], the dissociation constant of IgFLNa23-FilGAP peptide interaction would be ∼200 µM (0.2×∼1000), making it difficult to detect in pull down assays. Our data demonstrated that IgFLNa24 and the coiled-coil domain of FilGAP mediate dimerization of FLNa and FilGAP, respectively, and define their geometric arrangements and valencies, thereby increasing avidity of the FLNa-FilGAP complex. Moreover, FLNa hinge 2 (32aa residues) does not contribute to valency but defines the configurations that favor FilGAP binding (Figure 8). Electron micrographs of purified FLNa-FilGAP complex are consistent with the model.

Although no posttranslational modifications have been found so far in the FLNa-FilGAP binding sites, their higher order structure shown in Figure 8 suggests a possible mechanism for the regulation of this interaction by mechanical force. Our recent studies demonstrated that FLNa crosslinks F-actin using the conserved srABD and the second ABD that locates in rod segment 1 (rs1ABD), whereas the rod segment 2 is free from F-actin and accommodates the FilGAP interaction [11]. In addition, the geometry of IgFLNa23 defines its binding strength to FilGAP, suggesting that stretching or pushing of FLNa subunits attached to an F-actin network that is subjected to external or internal forces *in vivo* changes the geometry of two IgFLNa23 in FLNa dimer, hence affecting the avidity of the complex. The low stoichiometry of FLNa-FilGAP interaction determined *in vitro* and in cells (∼0.02, assessed from ref. [11], [12]) is in agreement with this idea. This mechanism may be in part responsible for FLNa's reported role in cellular mechanoprotection [19].

### Effects of FLNa-FilGAP interaction on cell spreading

Overexpression of wild-type FilGAP in cells has been shown to abolish integrin-mediated cell spreading, and a mutant FilGAP lacking its C-terminal 100 residues does not colocalize with FLNa in lamellae [12]. In this study we generated a FilGAP(V734Y) point mutant sufficient to disrupt FLNa-binding while keeping the coiled-coil domain intact. Consistent with a previous report, wild-type FilGAP suppressed lamella formation and cell spreading. Co-expression of EGFP-FLNa with wild-type FilGAP but not with the mutant FilGAP(V734Y) reduced the suppression effect, presumably because the mutant FilGAP unable to bind FLNa diffuses throughout cell and suppresses Rac-activity broadly. These results suggest that the binding of FilGAP to FLNa is important for proper spatiotemporal control of FilGAP functions.

### FilGAP is the first FLNa specific interaction partner

A mouse model and human developmental anomalies associated with FLNa mutations suggest that FLN isoforms have both redundant and distinct functions in cells and *in situ* due to their overlapping and unique distributions and partnering function [9], [20]–[24]. Here we found that FilGAP specifically interacts with FLNa, whereas FLNb and FLNc sequences are quite similar to FLNa and some binding partners interact with multiple filamin isoforms [25]. Specificity is conferred by single substitutions of amino acids at Ala2461 to Thr and Tyr2483 to His in FLNb and c, respectively.

### Disease-related FLNa mutations

Biological and rheological data suggest that two human disease-causing mutations (Δ7 and Δ3) impact on both signal transduction and mechanical deformation of cells. Since several binding partners have been reported to interact with the C-terminus of FLNa molecule, these mutations may perturb their interaction as well [1], [26]. However, our data demonstrate that these mutations completely disrupt FLNa interaction with FilGAP, suggesting that the Δ7 and Δ3 mutations at least perturb downstream of Rac, thereby affecting cell spreading. The F-actin-FLNa networks stiffen as they are strained [27], preventing large deformations that could threaten cellular integrity. Indeed, substantial mechanical stress induces apoptosis of FLNa-null cells [19]. The reduction of maximum stress of the F-actin-FLNa network by these two mutations, therefore, might affect cell survival under large mechanical stress.

The FLNa L2439M mutation does not affect FilGAP binding and rheological properties of F-actin/FLNa networks, which might explain why the presence of two different aberrant transcripts (L2439M and Δ7) in one patient exhibits overlap of PVNH and FMD. The results also suggest that some FLNa-binding partners interact with the strand A loop, where L2439M locates, instead of a groove of strand C and D of IgFLNa repeat which is the only binding interface characterized so far [14], [15].

In conclusion, by employing biochemical, computer modeling, and NMR analysis, which complement each other, we have depicted a higher-order structure of the FLNa-FilGAP complex. The structure explains how disease-causing mutations affect the complexing and mechanical properties of FLNa-actin networks, and suggests a novel mechanism of the regulation of FLNa-FilGAP interaction. Although we focused on the structure of the FLNa-FilGAP complex among over 50 binding partners identified thus far, this study reports first functional validation of FLNa-partner interactions in diseases using specific reagents informed by structural analysis.

## Materials and Methods

### Protein expression and purification

FLAG-tagged full-length FLNa was expressed using a Baculovirus Expression System (Invitrogen) in Sf9 insect cells and purified as previously described [11]. GST-His-FilGAP or MBP-His-FilGAP constructs were made by inserting PCR products of FilGAP domains to pGEX-4T-3-HT or pMALc-HT vectors [11], expressed in *E. coli*, and purified using glutathione, amylose, or Ni-NTA affinity columns as previously described [11]. Human full-length FilGAP fused to His-tag was expressed in Sf9 cells and purified using Ni-NTA affinity column. Tag-free FilGAP was prepared from His-FilGAP using TEV protease. His-tag FilGAP lacking 649–725 was expressed in *E. coli* (BL21(DE3)) using pET23-HTa vector [11] and purified by Ni-NTA affinity and Superose 6 gel filtration chromatography. GST-FLNa20-24 was expressed in *E. coli* using pGEX-4T-1 vector and purified by glutathione-Sepharose affinity chromatography. All the point or deletion mutants were generated using the QuickChange site-directed mutagenesis kit (Stratagene). His-IgFLNb23-24 was expressed in Sf9 cells and purified using Ni-NTA affinity column. After TEV cleavage, IgFLNb23-24 was gel filtered on Superdex200 10/300 column (GE healthcare). IgFLNc23-24 was prepared as previously described [28]. ^13^C^15^N-IgFLNa23 and ^15^N-IgFLNa23 mutants were produced in *E. Coli* as previously described [14]. For NMR studies the protein samples were buffered to pH 6.8 with 20 mM sodium phosphate. The samples also contained 150 mM NaCl, 1 mM DTT, 2 mM NaN_3_ and 7% (vol/vol) D_2_O.

### Structure determination

NMR experiments for structure determination were recorded on Varian INOVA 500 MHz, 600 MHz and 800 MHz spectrometers equipped with 5 mm inverse z-gradient triple resonance probe heads at 25°C. The structure determination was performed with 1 mM ^13^C^15^N-labeled IgFLNa23 sample. VNMR 6.1C software was used in spectrum acquisition and processing (Varian Inc., Palo Alto, CA). The triple-resonance experiments recorded for backbone resonance assignment were iHNCA and iHNCACB, HNCA, HN(CO)CA, HNCACB, HN(CO)CACB and HNCO [29]–[31]. The side-chain resonance assignment for aliphatic residues was done using CC(CO)NH and HCCH-COSY spectra. The aromatic side-chain resonances were assigned using the distance information from ^13^C-edited three-dimensional NOESY spectrum. Structural restraints were extracted from ^13^C- and ^15^N-edited three-dimensional NOESY spectra. The spectrum visualization software Sparky 3.110 was used in spectrum analysis [32]. Structure determination was done with the automatic NOE assignment mode of CYANA 2.1 software [33]. The set of 20 best structures selected after torsion angle dynamics calculation were refined with molecular dynamics in AMBER 8.0 using generalized Born implicit solvent model [34]. The quality of the refined structures was analyzed by using the WHAT_CHECK [35] and PROCHECK [36]_NMR programs. Ramachandran plot statistics for the ensemble of 20 refined structures: most favored, 91.1%; allowed, 8.0%; generously allowed, 0.6%; disallowed, 0.4%. The figures representing the protein structure were generated with MOLMOL [37] and PyMOL [38].

NMR experiments for structure determination were recorded on Varian INOVA 500 MHz, 600 MHz and 800 MHz spectrometers equipped with 5 mm inverse z-gradient triple resonance probe heads at 25°C. The structure determination was performed with 1 mM ^13^C^15^N-labeled IgFLNa23 sample. VNMR 6.1C software was used in spectrum acquisition and processing (Varian Inc., Palo Alto, CA). The triple-resonance experiments recorded for backbone resonance assignment were iHNCA and iHNCACB, HNCA, HN(CO)CA, HNCACB, HN(CO)CACB and HNCO [29]–[31]. The side-chain resonance assignment for aliphatic residues was done using CC(CO)NH and HCCH-COSY spectra. The aromatic side-chain resonances were assigned using the distance information from ^13^C-edited three-dimensional NOESY spectrum. Structural restraints were extracted from ^13^C- and ^15^N-edited three-dimensional NOESY spectra. The spectrum visualization software Sparky 3.110 was used in spectrum analysis [32]. Structure determination was done with the automatic NOE assignment mode of CYANA 2.1 software [33]. The set of 20 best structures selected after torsion angle dynamics calculation were refined with molecular dynamics in AMBER 8.0 using generalized Born implicit solvent model [34]. The quality of the refined structures was analyzed by using the WHAT_CHECK [35] and PROCHECK [36]_NMR programs. Ramachandran plot statistics for the ensemble of 20 refined structures: most favored, 91.1%; allowed, 8.0%; generously allowed, 0.6%; disallowed, 0.4%. The figures representing the protein structure were generated with MOLMOL [37] and PyMOL [38].

The folding state of IgFLNa23 mutants L2439M and M2474E was confirmed using ^15^N-HSQC spectra. The protein concentrations of the IgFLNa23-L2439M and IgFLNa23-M2474E NMR samples were 0.1 mM and 1 mM, respectively. The ^15^N-HSQC spectrum of IgFLNa23-M2474E was recorded on Varian INOVA 600 MHz spectrometer at 25°C. The ^15^N-HSQC spectrum of IgFLNa23-L2439M was recorded on Varian INOVA 600 MHz spectrometer equipped with 5 mm cryo probe at 25°C.

### FilGAP titration

The NMR titration experiments were performed with 0.5 mM ^13^C^15^N-labeled IgFLNa23 sample. The FilGAP peptide (FilGAP14: sequence ^723^EQFFSTFGELTVEP^736^) was purchased from EZBiolab Inc. A concentrated solution (5 mM) of FilGAP peptide was prepared in protein buffer and pH was adjusted to 6.8 with 1 M NaOH. The titration experiments were performed on Varian INOVA 500 MHz spectrometer equipped with 5 mm inverse z-gradient triple resonance probe head at 25°C.

### In silico modeling

The structure-based sequence alignment of IgFLNa23 and IgFLNa17 was made with Bodil [39]. The X-ray structure of IgFLNa17-GPIbα complex (pdb-code: 2BP3) [14] was used as a template structure to model the IgFLNa23 into peptide-binding conformation. The three-dimensional model of IgFLNa23-FilGAPC32 complex was constructed with Modeller 9v1 [40]. Coiled-coil domain structure was predicted by EMBnet COILS (http://www.ch.embnet.org/software/COILS_form.html). Sequence alignment was generated using ClustalW program (http://www.ebi.ac.uk/Tools/clustalw2/index.html).

### FLAG-FLNa pull-down assay

Various concentration of GST-His- or MBP-His-FilGAP constructs were incubated with 10 µl of FLAG-specific mAb M2 agarose (50% vol/vol slurry, Sigma) in the presence or absence of 25 nM FLAG-FLNa and in binding buffer (50 mM Tris-HCl, 150 mM NaCl, 0.1% (wt/vol) Triton X-100, 0.1 mM β-mercaptoethanol, 0.1 mM EGTA, pH 7.4; 400 µl) for 1 h at 25°C. The beads were sedimented and washed 3 times with binding buffer. Proteins bound to the beads were solublized in SDS sample buffer and separated by 9.5% or 12.5% (wt/vol) SDS-PAGE followed by immunoblotting using rabbit polyclonal antibodies (pAbs) against GST (Sigma), MBP (New England Biolabs), or FilGAP [12] or mouse mAb against His conjugated with horse radish peroxidase.

### In vitro immunoprecipitation

Purified FilGAP (50 nM) were incubated with increasing amounts of either FLNa or FLNb in binding buffer and co-immunoprecipitated with mouse mAb to FLNa (5 µg each of 3-14 and 4-3) [11] or FLNb (10 µg of 1-11c) [14] immobilized on 20 µl of GammaBind G-Sepharose beads (GE healthcare) for overnight at 4°C. The beads were sedimented and washed 3 times with binding buffer. Proteins bound to the beads were solublized in SDS sample buffer and separated by 9.5% (wt/vol) SDS-PAGE followed by immunoblotting using rabbit pAbs to FilGAP.

### MBP-FilGAP pull-down assay

FLNa or its truncates were incubated with 30 µl of amylose resin (50% vol/vol slurry, New England Biolabs) coated with various concentration of MBP-FilGAP 649–748 in the binding buffer for 1 h at 25°C. The beads were sedimented and washed 3 times with binding buffer. Proteins bound to the beads were solublized in SDS sample buffer and separated by 9% or 12.5% (wt/vol) SDS-PAGE. Polypeptides in the gels were visualized by Coomassie brilliant blue (CBB) staining or immunoblotting.

### FLNa and FilGAP mammalian expression vectors

The ClaI/XbaI FLNa fragment of pEGFP-FLNa [14] vector was cloned into pBluescript II SK vector, mutated using the QuickChange site-directed mutagenesis kit, and then cloned back to pEGFP-FLNa or pREP4-FLNa[41] vectors. The pCDNA5-HA-FilGAP [12] was directly mutated using the QuickChange site-directed mutagenesis kit.

### Cell culture and immunoprecipitation

Human melanoma FLNa-deficient cell lines (M2) were transfected with the pCDNA5-HA-FilGAP (wild-type or V734Y) and the pREP4-FLNa (wild-type or M2474E) using FuGENE 6 as described by the manufacturer's instructions (Roche applied science). Forty-eight hours later, the cells were rinsed three times with PBS and lysed in 50 mM Tris-HCl, 150 mM NaCl, 1% (wt/vol) TritonX-100, 1 mM β-mercaptoethanol, 5 mM EGTA, 10 ìg/ml leupeptin, pepstatinA, aprotinin, 2 mM PMSA, 0.1 mg/ml DNAseI. The cell lysates were pre-cleared and subjected to immunoprecipitation with antibodies to FLNa [42] to precipitate transfected FLNa. Bound protein was detected by western blotting using mouse mAbs to FLNa for FLNa and rat mAb to HA (Roche applied science) for FilGAP.

### Cell spreading assay

M2 cell lines were transfected with the pCDNA5-HA-FilGAP (wild-type or V734Y) and/or pEGFP-FLNa [14] using FuGENE 6. After 48 h, cells were trypsinized and plated on coverslips coated with 10 ìg/ml human fibronectin (Sigma) and fixed at indicated time with 3.7% (wt/vol) formalin in PBS for 20 min. The fixed cells were permeabilized with 0.5% (wt/vol) Triton X-100 in PBS for 5 min and blocked with 0.2% (wt/vol) BSA, 0.1% (wt/vol) sodium azide and 0.1% (wt/vol) Triton X-100 in PBS for 1 h. HA-FilGAP was stained with mouse mAb to HA (HA-7, Sigma) at 1∶500 dilution and mouse IgG-specific goat pAbs conjugated with Alexa-568 (Invitrogen) at 1∶400 dilution. Spread cells were examined as described previously [12].

### Gel-filtration Analysis

Purified FLNa constructs were loaded on a Superose 6 10/300 GL column (GE healthcare) and eluted at a flow rate of 0.4 ml/min in 20 mM Tris-HCl, pH 7.4, 150 mM NaCl, 0.5 mM EGTA, 0.5 mM β-mercaptoethanol at 4°C. Molecular size standards used were thyroglobulin (669 kDa), ferritin (440 kDa), aldolase (158 kDa), conalbumin (75 kDa), and ovalbumin (43 kDa).

### Rheology

To probe the mechanical properties of F-actin-/FLNa networks, we employed a stress-controlled mechanical shear rheometer (Bohlin, Malvern Instruments) equipped with a 40-mm parallel plate geometry and a gap of 100 µm as previously described [27]. Briefly, 12 µM monomeric actin was mixed with 0.06 µM wild-type or mutant FLNa and polymerization buffer (total volume 150 µL) and immediately placed between the stainless steel plates of the rheometer. The sample was allowed one hour to polymerize before measuring the dynamic viscoelastic moduli.

Gel points were measured as previously described [43].

### Accession codes

The resonance assignments and the distance restraints used in IgFLNa23 NMR structure calculation have been deposited in BioMagResBank under accession number 15777. The coordinates of the structure ensemble have been deposited in Protein Data Bank under accession code 2k3t.

## Supporting Information

Figure S1 Physical properties of the recombinant proteins. (A) Coomassie blue stain of 8–16% gradient Tris-Glycine SDS-PAGE of each purified recombinant protein (0.5 mg). (B) Gel filtration analysis of FilGAP constructs on Superose6 10/300 GL. Apparent molecular weight (Mr) were determined from elution volumes by comparing to those of throglobulin, ferritin, aldorase, conalbulin, ovalbumin, and ribonuclease A (left to right).
(8.97 MB TIF)

Figure S2 Electron micrographs of the purified FLNa and its mutants. Structure of full-length FLNa and mutant FLAG-FLNa were determined by low angle rotary shadowing of molecules sprayed onto mica and dried under vacuum.
(3.72 MB TIF)

Figure S3 NMR titration of FilGAP peptide to IgFLNa23. (A) 15N-HSQC spectrum of IgFLNa23+660%FilGAP14 (blue) superimposed on 15N-HSQC spectrum of IgFLNa23 (red). (B) Chemical shift changes in the 15N-HSQC spectrum of IgFLNa23 upon addition of 6.6-fold excess of FilGAP14 as a function of sequence. Black = overlapping signal which could not be traced reliably. Light gray = signal has broadened beyond detection or it has divided into multiple peaks. The chemical shift difference was set to 0.22 ppm.
(6.83 MB TIF)

Figure S4 Comparison of IgFLNa23, IgFLNa17 and IgFLNa17-GPIbα complex. Based on the X-ray structure of IgFLNa17-GPIbα complex (pink ribbon), it was possible to build a model for IgFLNa23-FilGAPC32 complex (not shown) where the IgFLNa23 is in such conformation that it can bind the FilGAPC32 peptide. When compared to the NMR structure of IgFLNa23 (gray ribbon) and IgFLNa17 (cyan ribbon), peptide binding seems to force the C and D strands further away from each other, especially between the N-terminus of C strand and C-terminus of D-strand. The binding of FilGAP peptide to IgFLNa23 reflects at the structural level particularly to the conformation of the Y2483 side-chain that is pushed aside to allow the binding of peptide binding. Chemical shift changes of Y2483 are seen in the FilGAP peptide NMR titration experiments (Figure 3 and Figure S3).
(6.29 MB TIF)

Figure S5 Chemical shift changes induced to the 15N-HSQC spectrum of IgFLNa23 by M2474E mutation. (A) Superimposition of the 15N-HSQC spectra of IgFLNa23 (red) and M2474E IgFLNa23 (blue). (B) Chemical shift difference as a function of sequence. Black = mutated residue, light gray = signal has shifted too much to be identified without complete reassignment. The chemical shift difference was set to 0.4 ppm. (C) Chemical shift differences exceeding 0.1 ppm mapped on the structure of IgFLNa23. The mutated residue is indicated with stick model.
(9.30 MB TIF)

Figure S6 Rheological properties of 12 µM F-actin networks cross-linked with 0.06 µM purified FLNa and its mutants. (A) The linear elastic moduli, G′ (closed circles), and viscous moduli, G′ (open circles), as a function of frequency. (B) The nonlinear elastic moduli as a function of strain, measured at f = 0.1 Hz. (C) The nonlinear elastic moduli as a function of applied stress, measured at f = 0.1 Hz.
(7.49 MB TIF)

Figure S7 Chemical shift changes induced to the 15N-HSQC spectrum of IgFLNa23 by L2439M mutation. (A) Superimposition of the 15N-HSQC spectra of IgFLNa23 (red) and L2439M IgFLNa23 (blue). (B) Chemical shift difference as a function of sequence. Black = mutated residue, light gray = signal has shifted too much to be identified without complete reassignment. The chemical shift difference was set to 0.4 ppm. (C) Chemical shift differences exceeding 0.1 ppm mapped on the structure of IgFLNa23. The mutated residue is indicated with stick model.
(9.27 MB TIF)

Figure S8 Electron micrographs of the purified FLNa and FilGAP. Rotary shadowed images of purified FilGAP (top), FLNa (second from the top), the FLNa/FilGAP complex (third from the top), mutant FLNaM2474E mixed with FilGAP (the second from the bottom), and FLNaM2474E (bottom). FLNa and FilGAP were mixed at a 1FLNa∶20FilGAP ratio for 1 h end-over-end at room temperature, diluted 1∶4 in 67% glycerol to a final protein concentration of 25 µg/ml (50% glycerol), and sprayed onto mica as previously described1. About 26% (26 / 100) of wild-type FLNa molecules were complexed with FilGAP, whereas FLNaM2474E mutant molecules were not.
(3.60 MB TIF)

Table S1 NMR and refinement statistics for IgFLNa23.
(1.69 MB TIF)
