# Supplementary figures and images for: Molecular Basis of Filamin A-FilGAP Interaction and Its Impairment in Congenital Disorders Associated with Filamin A Mutations
Source: PLoS One. 2009 Mar 18;4(3):e4928. doi: 10.1371/journal.pone.0004928 (PMC2654154; doi:10.1371/journal.pone.0004928)

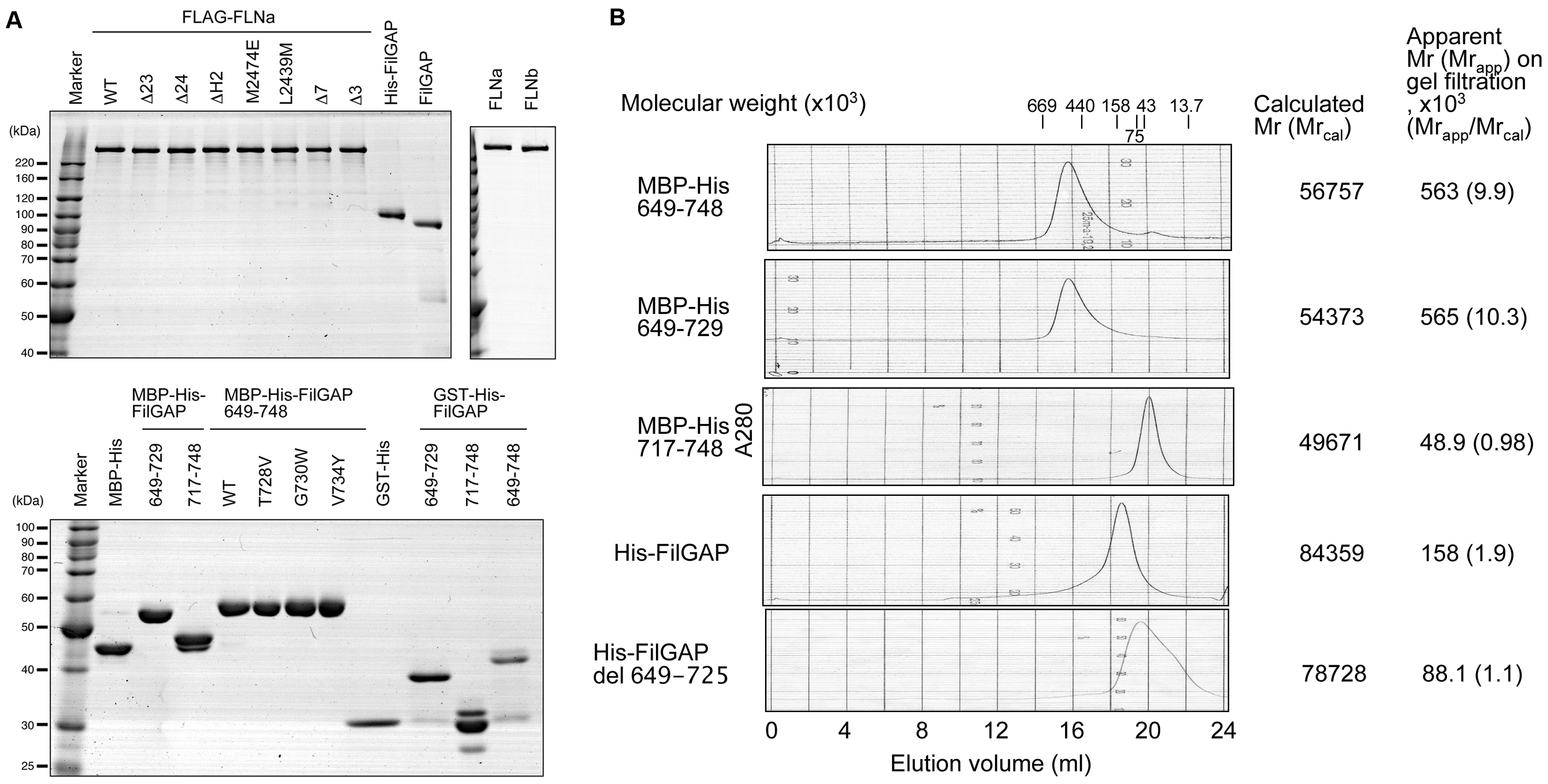

Supplement: Figure S1 — Physical properties of the recombinant proteins. (A) Coomassie blue stain of 8–16% gradient Tris-Glycine SDS-PAGE of each purified recombinant protein (0.5 mg). (B) Gel filtration analysis of FilGAP constructs on Superose6 10/300 GL. Apparent molecular weight (Mr) were determined from elution volumes by comparing to those of throglobulin, ferritin, aldorase, conalbulin, ovalbumin, and ribonuclease A (left to right). (8.97 MB TIF) [file pone.0004928.s001.tif]

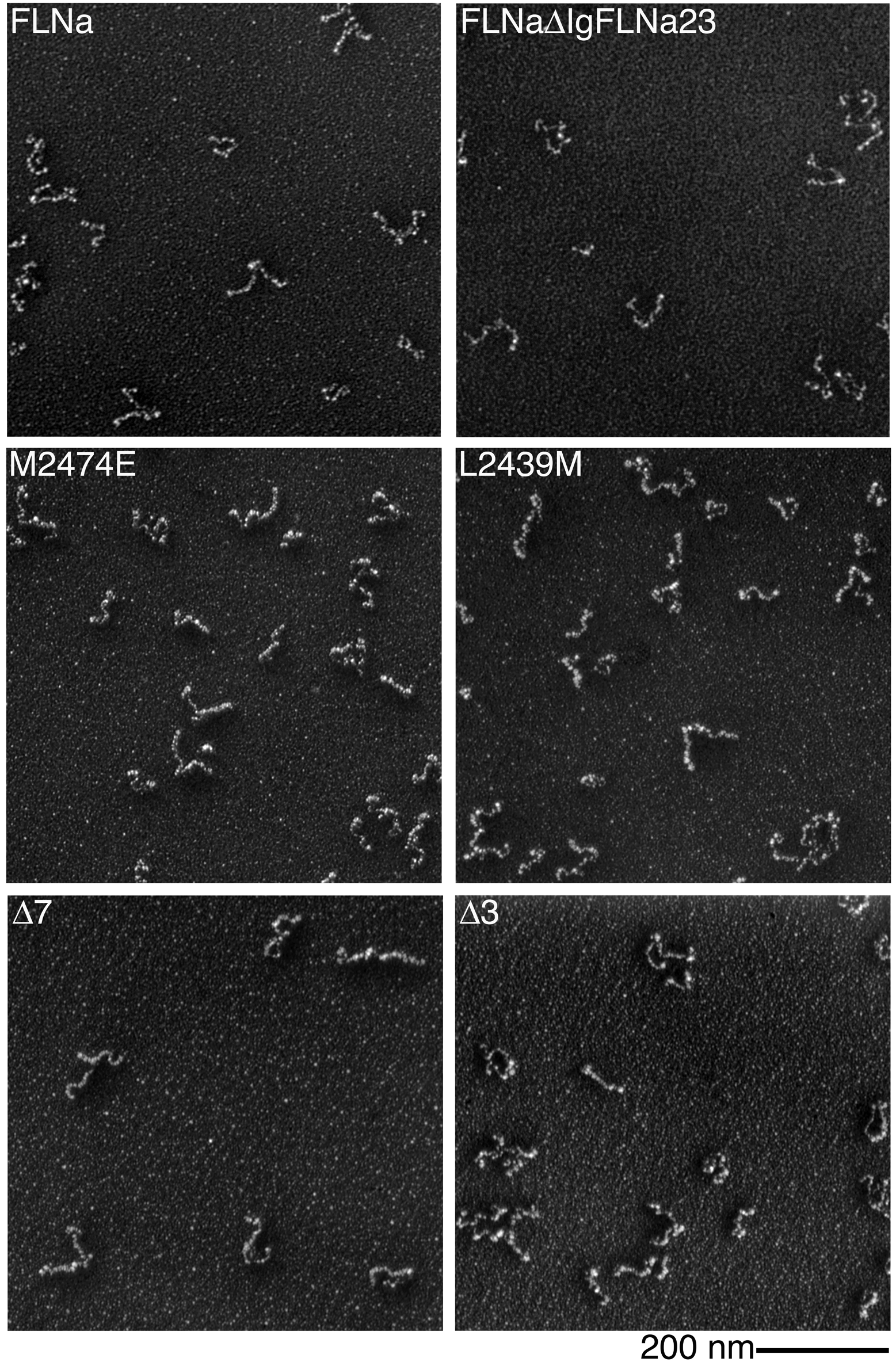

Supplement: Figure S2 — Electron micrographs of the purified FLNa and its mutants. Structure of full-length FLNa and mutant FLAG-FLNa were determined by low angle rotary shadowing of molecules sprayed onto mica and dried under vacuum. (3.72 MB TIF) [file pone.0004928.s002.tif]

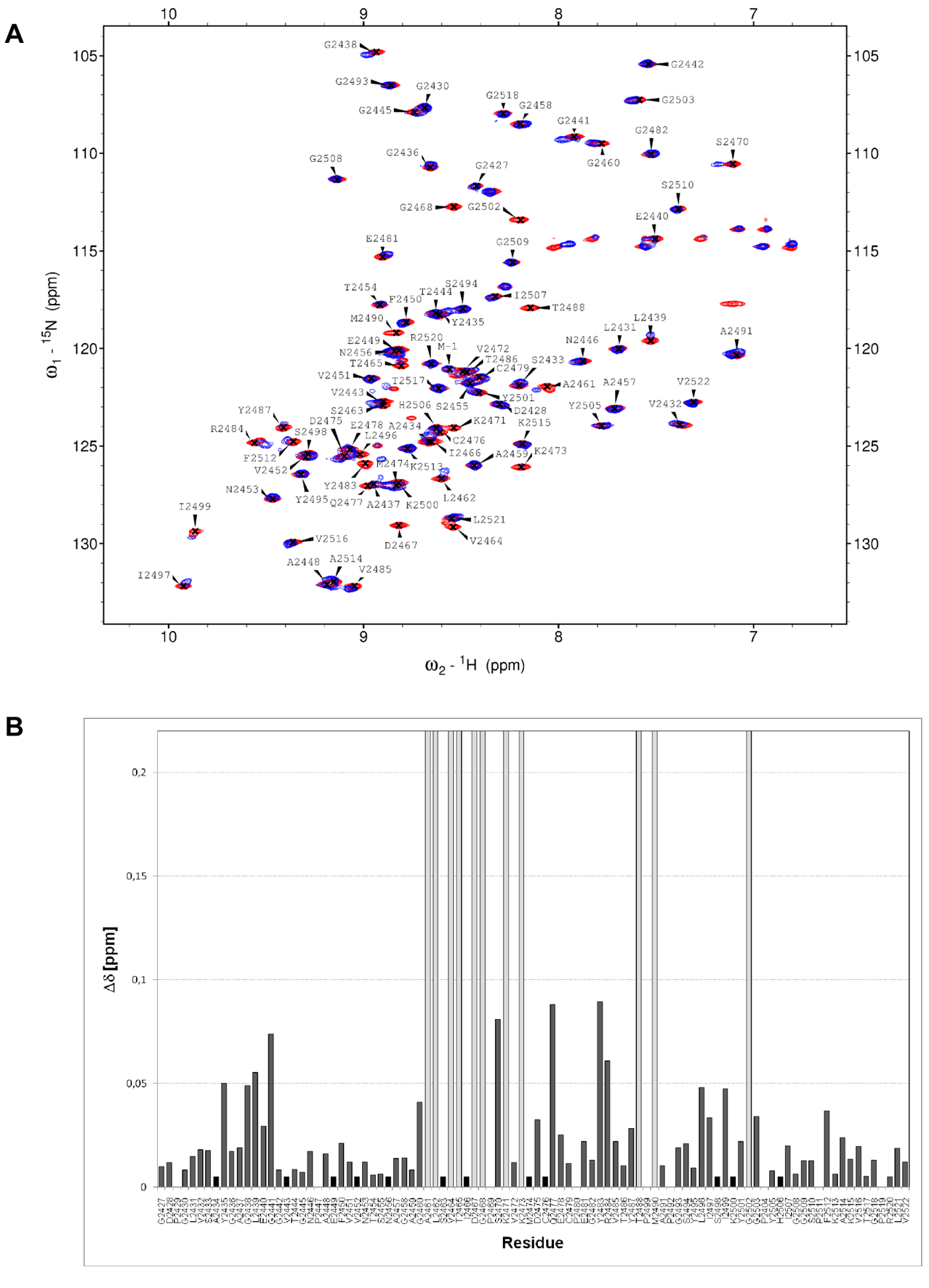

Supplement: Figure S3 — NMR titration of FilGAP peptide to IgFLNa23. (A) 15N-HSQC spectrum of IgFLNa23+660%FilGAP14 (blue) superimposed on 15N-HSQC spectrum of IgFLNa23 (red). (B) Chemical shift changes in the 15N-HSQC spectrum of IgFLNa23 upon addition of 6.6-fold excess of FilGAP14 as a function of sequence. Black = overlapping signal which could not be traced reliably. Light gray = signal has broadened beyond detection or it has divided into multiple peaks. The chemical shift difference was set to 0.22 ppm. (6.83 MB TIF) [file pone.0004928.s003.tif]

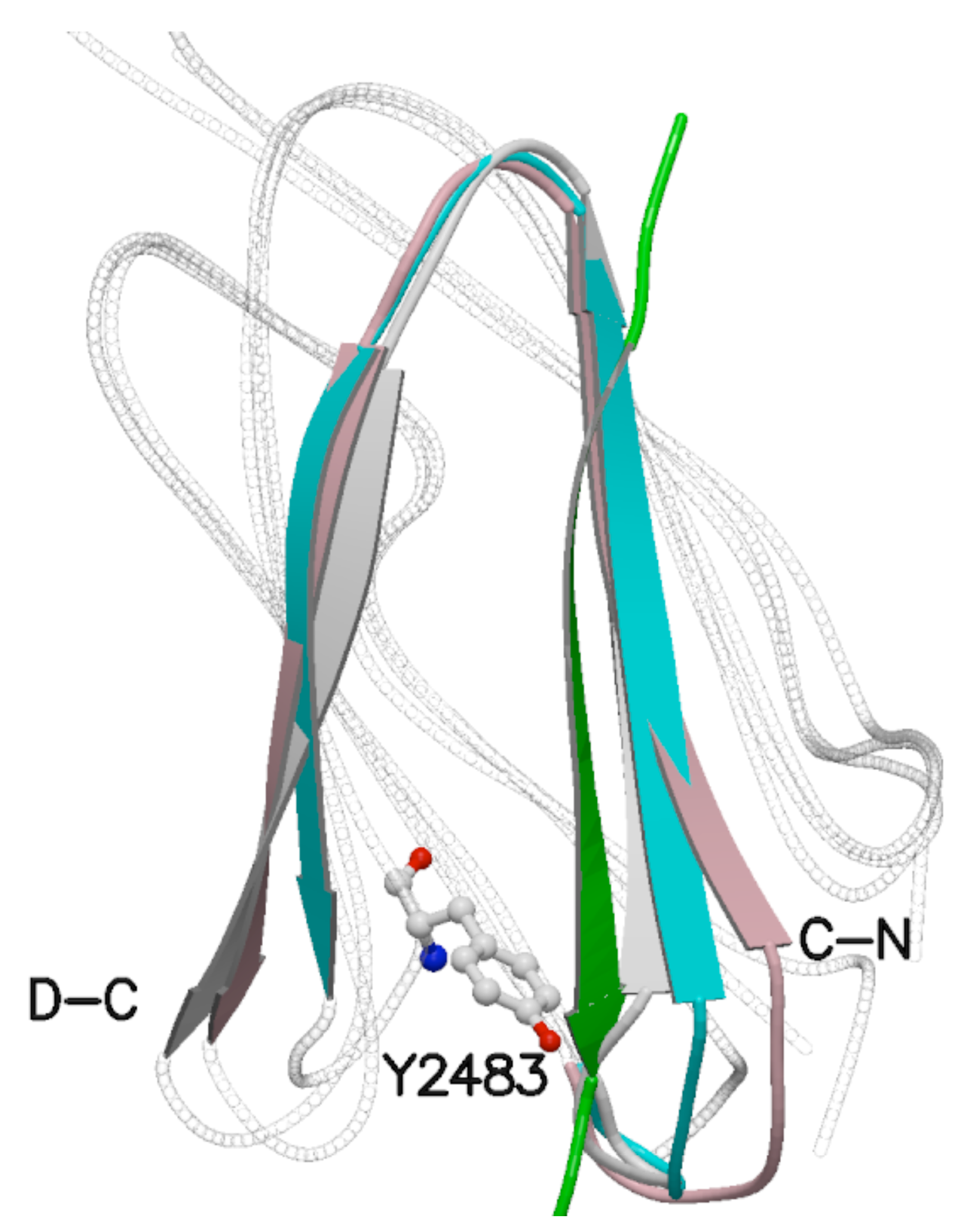

Supplement: Figure S4 — Comparison of IgFLNa23, IgFLNa17 and IgFLNa17-GPIbα complex. Based on the X-ray structure of IgFLNa17-GPIbα complex (pink ribbon), it was possible to build a model for IgFLNa23-FilGAPC32 complex (not shown) where the IgFLNa23 is in such conformation that it can bind the FilGAPC32 peptide. When compared to the NMR structure of IgFLNa23 (gray ribbon) and IgFLNa17 (cyan ribbon), peptide binding seems to force the C and D strands further away from each other, especially between the N-terminus of C strand and C-terminus of D-strand. The binding of FilGAP peptide to IgFLNa23 reflects at the structural level particularly to the conformation of the Y2483 side-chain that is pushed aside to allow the binding of peptide binding. Chemical shift changes of Y2483 are seen in the FilGAP peptide NMR titration experiments (Figure 3 and Figure S3). (6.29 MB TIF) [file pone.0004928.s004.tif]

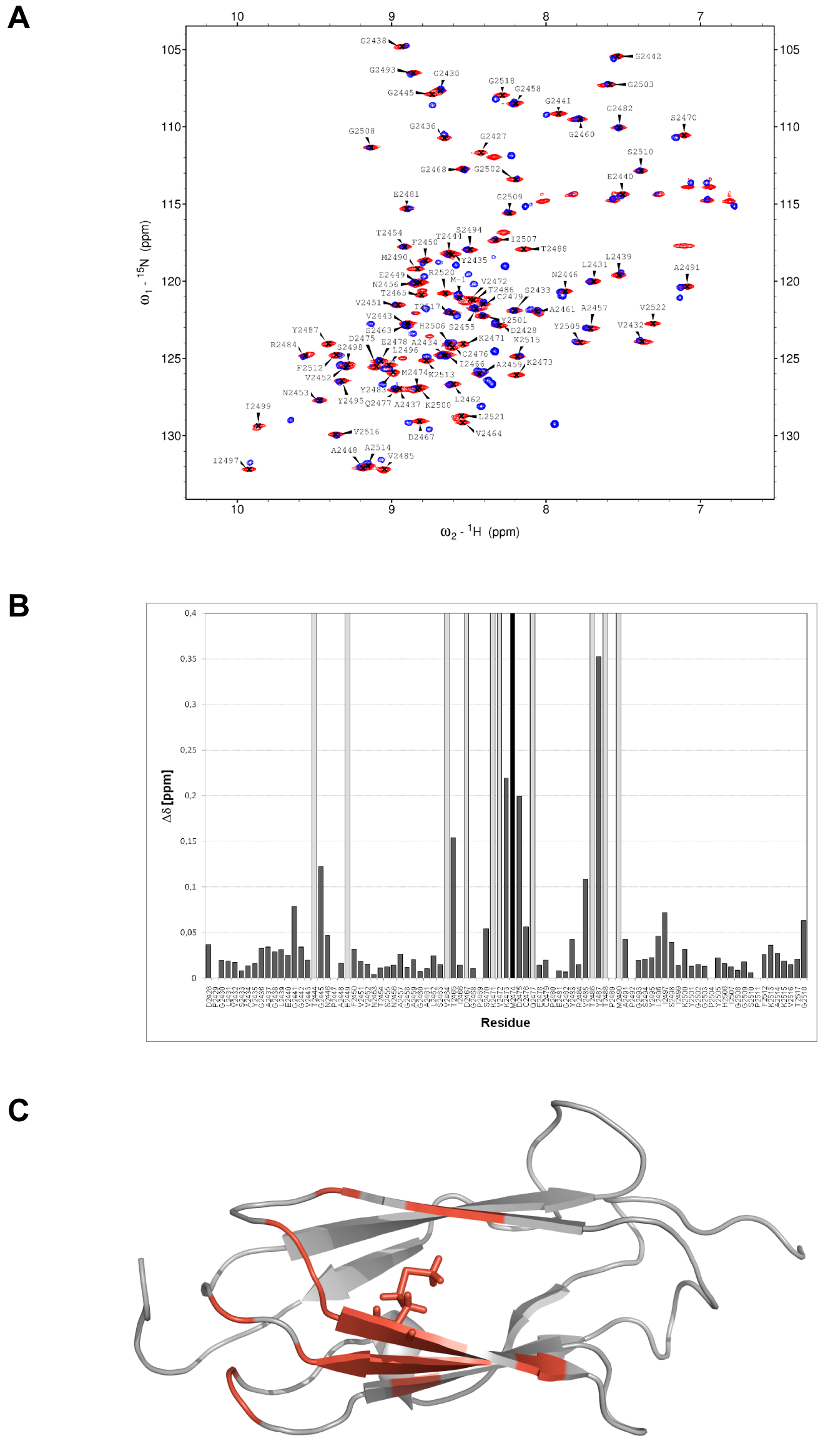

Supplement: Figure S5 — Chemical shift changes induced to the 15N-HSQC spectrum of IgFLNa23 by M2474E mutation. (A) Superimposition of the 15N-HSQC spectra of IgFLNa23 (red) and M2474E IgFLNa23 (blue). (B) Chemical shift difference as a function of sequence. Black = mutated residue, light gray = signal has shifted too much to be identified without complete reassignment. The chemical shift difference was set to 0.4 ppm. (C) Chemical shift differences exceeding 0.1 ppm mapped on the structure of IgFLNa23. The mutated residue is indicated with stick model. (9.30 MB TIF) [file pone.0004928.s005.tif]

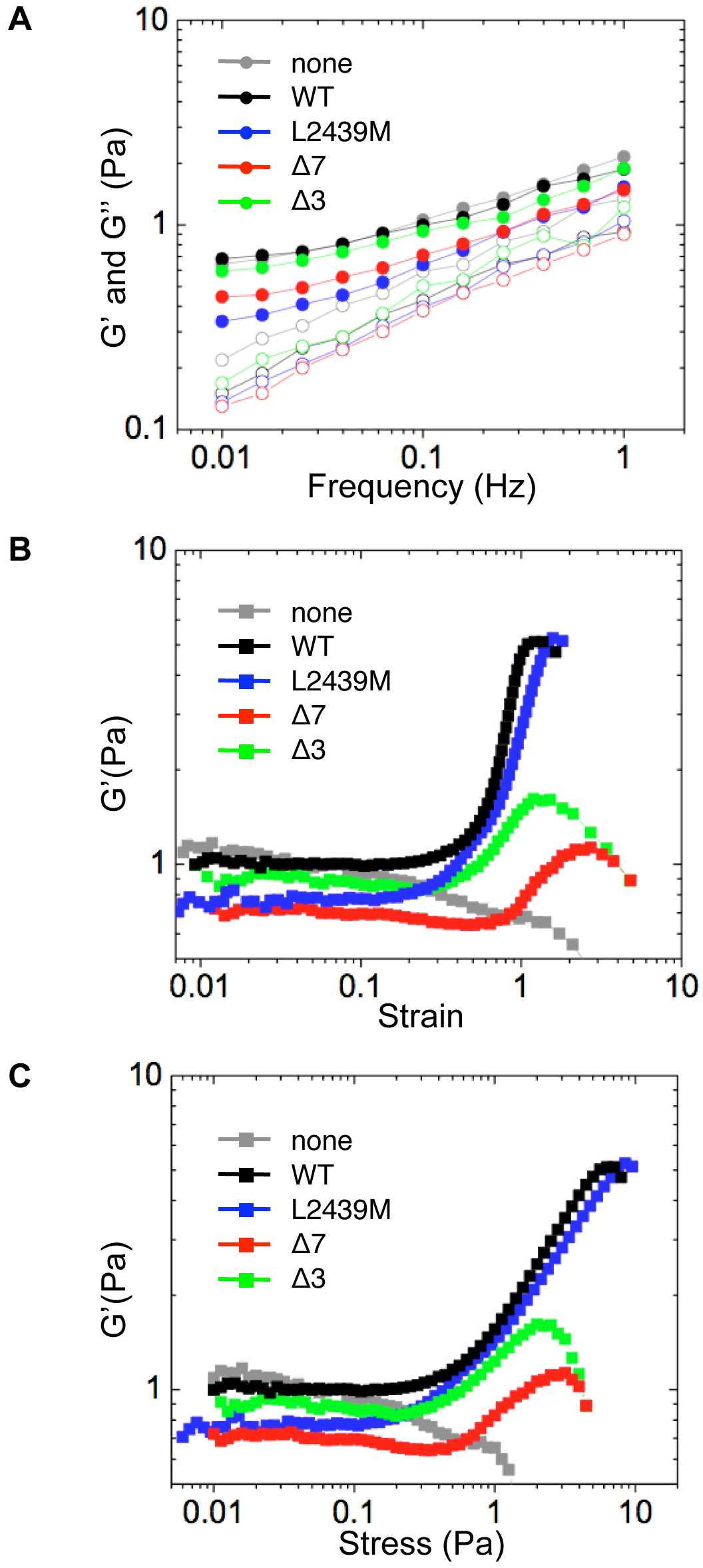

Supplement: Figure S6 — Rheological properties of 12 µM F-actin networks cross-linked with 0.06 µM purified FLNa and its mutants. (A) The linear elastic moduli, G′ (closed circles), and viscous moduli, G′ (open circles), as a function of frequency. (B) The nonlinear elastic moduli as a function of strain, measured at f = 0.1 Hz. (C) The nonlinear elastic moduli as a function of applied stress, measured at f = 0.1 Hz. (7.49 MB TIF) [file pone.0004928.s006.tif]

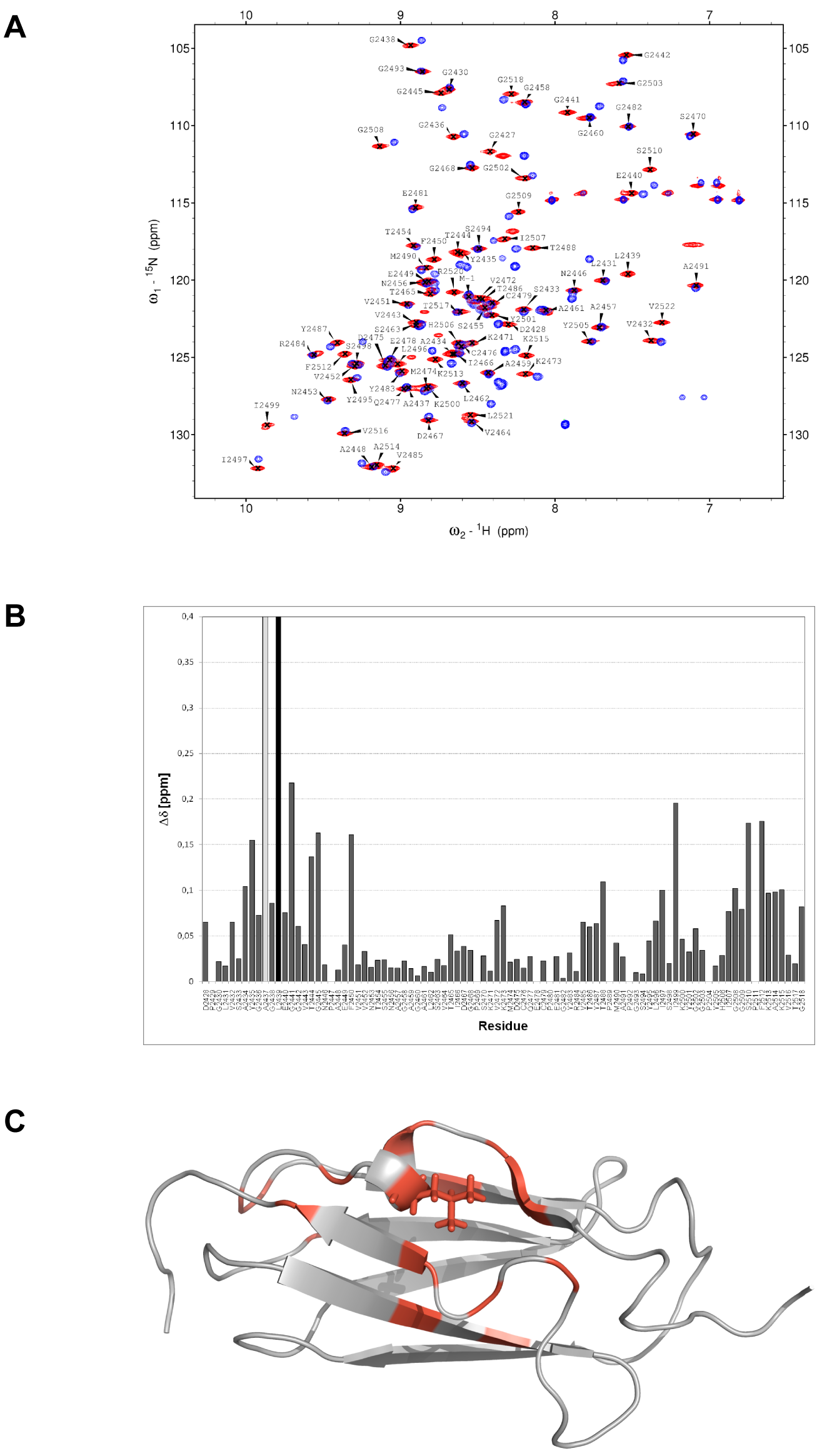

Supplement: Figure S7 — Chemical shift changes induced to the 15N-HSQC spectrum of IgFLNa23 by L2439M mutation. (A) Superimposition of the 15N-HSQC spectra of IgFLNa23 (red) and L2439M IgFLNa23 (blue). (B) Chemical shift difference as a function of sequence. Black = mutated residue, light gray = signal has shifted too much to be identified without complete reassignment. The chemical shift difference was set to 0.4 ppm. (C) Chemical shift differences exceeding 0.1 ppm mapped on the structure of IgFLNa23. The mutated residue is indicated with stick model. (9.27 MB TIF) [file pone.0004928.s007.tif]

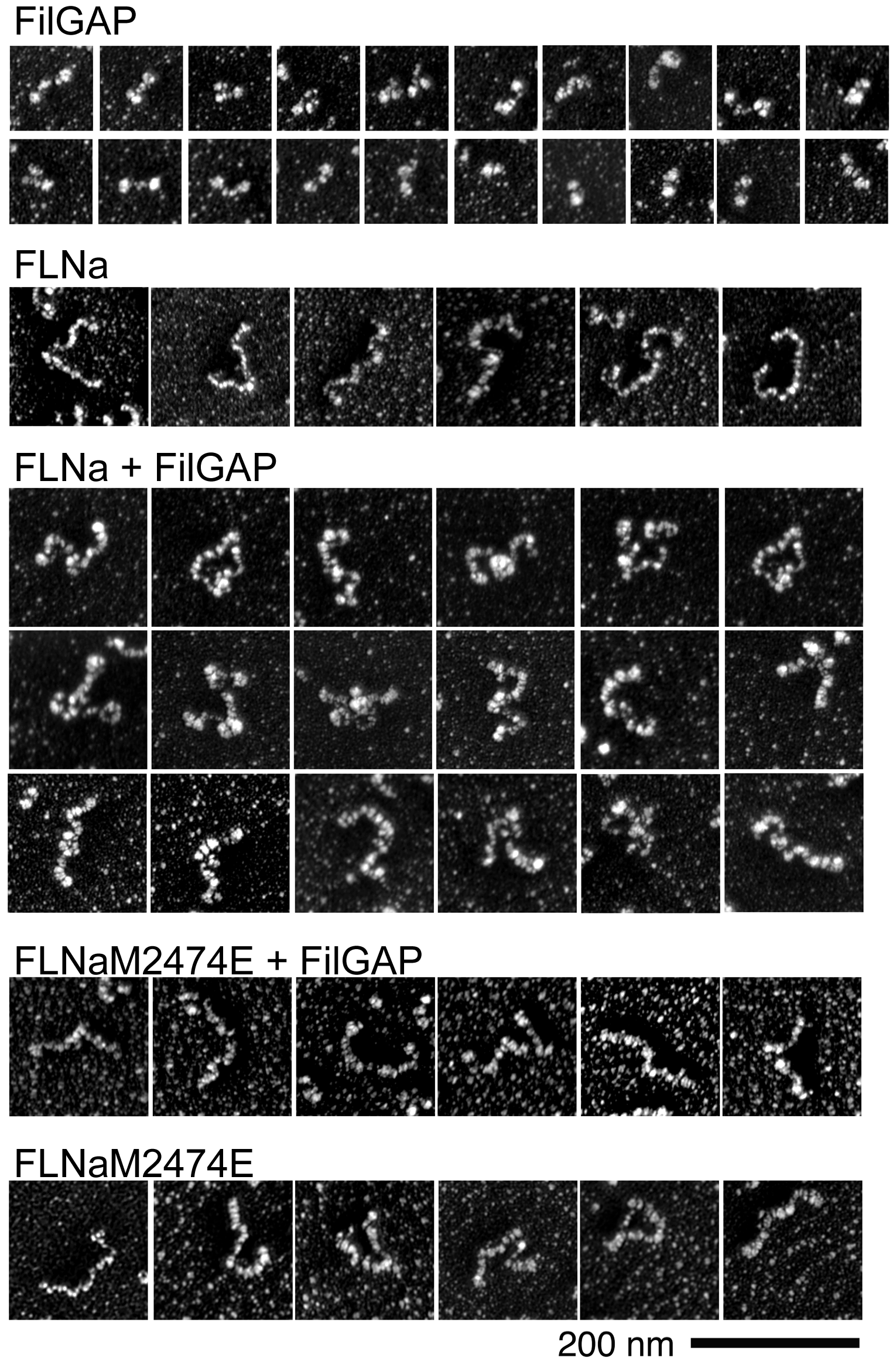

Supplement: Figure S8 — Electron micrographs of the purified FLNa and FilGAP. Rotary shadowed images of purified FilGAP (top), FLNa (second from the top), the FLNa/FilGAP complex (third from the top), mutant FLNaM2474E mixed with FilGAP (the second from the bottom), and FLNaM2474E (bottom). FLNa and FilGAP were mixed at a 1FLNa∶20FilGAP ratio for 1 h end-over-end at room temperature, diluted 1∶4 in 67% glycerol to a final protein concentration of 25 µg/ml (50% glycerol), and sprayed onto mica as previously described1. About 26% (26 / 100) of wild-type FLNa molecules were complexed with FilGAP, whereas FLNaM2474E mutant molecules were not. (3.60 MB TIF) [file pone.0004928.s008.tif]

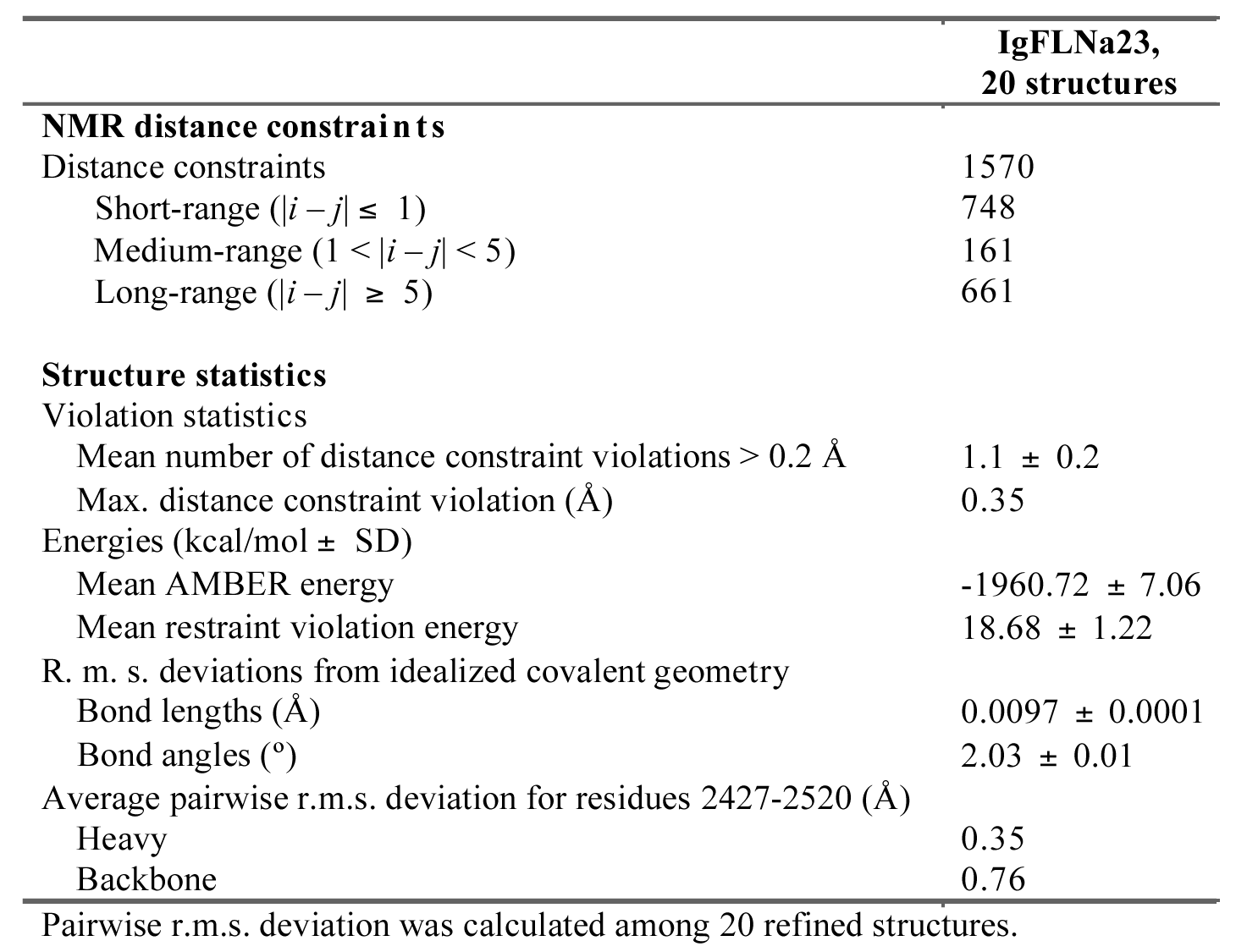

Supplement: Table S1 — NMR and refinement statistics for IgFLNa23. (1.69 MB TIF) [file pone.0004928.s009.tif]
